# Tissue Specific Roles for the Ribosome Biogenesis Factor Wdr43 in Zebrafish Development

**DOI:** 10.1371/journal.pgen.1004074

**Published:** 2014-01-30

**Authors:** Chengtian Zhao, Viktoria Andreeva, Yann Gibert, Melissa LaBonty, Victoria Lattanzi, Shubhangi Prabhudesai, Yi Zhou, Leonard Zon, Kathleen L. McCann, Susan Baserga, Pamela C. Yelick

**Affiliations:** 1 Department of Oral and Maxillofacial Pathology, Division of Craniofacial and Molecular Genetics, Tufts University, Boston, Massachusetts, United States of America; 2 Institute of Evolution and Marine Biodiversity, Ocean University of China, Qingdao, China; 3 Children's Hospital Boston and Harvard Medical School, Boston, Massachusetts, United States of America; 4 Department of Genetics, Yale School of Medicine, New Haven, Connecticut, United States of America; 5 Departments of Molecular Biophysics & Biochemistry and Therapeutic Radiology, Yale School of Medicine, New Haven, Connecticut, United States of America; USC Keck School of Medicine, United States of America

## Abstract

During vertebrate craniofacial development, neural crest cells (NCCs) contribute to most of the craniofacial pharyngeal skeleton. Defects in NCC specification, migration and differentiation resulting in malformations in the craniofacial complex are associated with human craniofacial disorders including Treacher-Collins Syndrome, caused by mutations in *TCOF1*. It has been hypothesized that perturbed ribosome biogenesis and resulting p53 mediated neuroepithelial apoptosis results in NCC hypoplasia in mouse *Tcof1* mutants. However, the underlying mechanisms linking ribosome biogenesis and NCC development remain poorly understood. Here we report a new zebrafish mutant, *fantome (fan)*, which harbors a point mutation and predicted premature stop codon in zebrafish *wdr43*, the ortholog to yeast UTP5. Although *wdr43* mRNA is widely expressed during early zebrafish development, and its deficiency triggers early neural, eye, heart and pharyngeal arch defects, later defects appear fairly restricted to NCC derived craniofacial cartilages. Here we show that the C-terminus of Wdr43, which is absent in *fan* mutant protein, is both necessary and sufficient to mediate its nucleolar localization and protein interactions in metazoans. We demonstrate that Wdr43 functions in ribosome biogenesis, and that defects observed in *fan* mutants are mediated by a p53 dependent pathway. Finally, we show that proper localization of a variety of nucleolar proteins, including TCOF1, is dependent on that of WDR43. Together, our findings provide new insight into roles for Wdr43 in development, ribosome biogenesis, and also ribosomopathy-induced craniofacial phenotypes including Treacher-Collins Syndrome.

## Author Summary

Here, we describe the identification and characterization of a novel zebrafish craniofacial mutant, *fantome (fan)*, caused by a point mutation in the *wdr43* gene. Although previously characterized as UTP5 in yeast, a nucleolar protein functioning in ribosome biogenesis, here we show that Wdr43 also regulates early zebrafish development, including NCC specification and differentiation. Mutations in nucleolar proteins have been found to be causative for a variety of human craniofacial syndromes including Treacher-Collins Syndrome (TCS), often caused by mutations in *TCOF1*, which also plays important roles in ribosome biogenesis. However, the underlying mechanisms linking ribosomal biogenesis and NCC specification and differentiation into pharyngeal arch cartilages remains poorly understood. Here we describe the *fan/wdr43* mutant phenotype, and present functional characterizations of Wdr43 in craniofacial development. We show that WDR43 is required for the proper nucleolar localization of a variety of nucleolar proteins, including TCOF1/Treacle. These studies provide new insight into ribosomal protein function in early zebrafish development, with focus on NCC derived craniofacial development, as a model for human craniofacial neurocristopathies.

## Introduction

Neural crest cells (NCCs), a transient cell type that is unique to vertebrates, originate from the dorsal aspect of the neural tube during embryogenesis. After undergoing an epithelial-to-mesenchymal transition (EMT), NCCs migrate along well defined pathways, and eventually inhabit peripheral destinations where they differentiate into diverse derivatives, including melanocytes, craniofacial cartilage and bone, smooth muscle, and neuronal lineages. In the head region, cranial neural crest cells (CNCC) give rise to nearly all craniofacial structures, including the facial skeleton and the vast majority of facial connective tissues [1], [2]. Defects in CNCC development are associated with craniofacial malformations, one of the most common of human birth defects [3].

Treacher-Collins syndrome (TCS), an autosomal dominant congenital disorder of craniofacial development, characterized by mandibulofacial dysostosis including cleft palate and hypoplasia of the facial bones, is most commonly associated with mutations in the *TCOF1* gene [4]. Treacle, the protein encoded by the *TCOF1* gene, is a nucleolar phosphoprotein [5], [6] that plays a key role in ribosome biogenesis via involvement in both rDNA methylation and rRNA transcription [7]–[10]. Extensive research in the mouse model has shown that mutations in *Tcof1* disrupt ribosome biogenesis, resulting in impaired proliferation and subsequent apoptosis of neuroepithelial and NCC precursors, which in turn results in reduced numbers of NCCs migrating into the developing craniofacial complex [10]. Interestingly, inhibition of p53 function can rescue craniofacial abnormalities in mouse *Tcof1* mutants, without rescuing ribosome biogenesis defects [11].

The question of how ribosome biogenesis defects can preferentially affect NCC proliferation and differentiation remains to be elucidated. In eukaryotic cells, ribosome biogenesis begins with the transcription of rRNA from rDNA located in the nucleolus, the most prominent visible structure in the nucleus. Ribosome biogenesis is extremely complex, requiring the accurate processing of pre-rRNAs into four different ribosomal RNAs (28S, 18S, 5S and 5.8S in vertebrates) and complex formation with about 80 constituent ribosomal proteins. In addition, more than 200 nucleolar ribosome biogenesis factors are required to complete the entire ribosome biogenesis process. All ribosomal RNAs, except the 5S rRNA, are initially transcribed as a 47S polycistronic precursor, which subsequently becomes cleaved, folded and modified into the 28S, 18S and 5.8S mature rRNAs prior to being incorporated into functional ribosomes [12], [13].

The cleavage and modification of rRNA is directed by small nucleolar RNAs (snoRNAs). U3, one of the most extensively studied snoRNA, is an essential component of the small subunit (SSU) processome, a large ribonucleoprotein (RNP) complex that is required for the maturation of the 18S rRNA and formation of the ribosomal small subunit (40S) [14]. The SSU processome can be further subdivided to three sub-complexes, UTPB, UTPC and UTPA/t-Utp [14]. The t-Utp complex contains seven proteins (Utp4, Utp5, Utp8, Utp9, Utp10, Utp15 and Utp17) [15], [16]. In yeast, depletion of individual t-Utp members commonly is associated with both pre-rRNA synthesis and processing defects [14], [16], [17], although another group reported that the t-Utp subcomplex plays a role in pre-rRNA stabilization rather than transcription [18]. Although the functions of t-Utp components appear to be conserved in eukaryotes, some human UTP orthologs have not yet been identified [19], indicating that Utp proteins in higher eukaryotes may have evolved specific functions. Recently, a new metazoan specific protein, NOL11, has been characterized as a hUTP4 interacting partner via yeast two hybrid (Y2H) analysis [20].

Although ubiquitously expressed in virtually all eukaryotic cells, mutations in ribosome biogenesis proteins often result in tissue-specific developmental defects [21]. For example, hUTP4/Cirhin is associated with North American Indian childhood cirrhosis (NAIC) [22]. Mutation of zebrafish Bap28, the ortholog of human UTP10, results in excess apoptosis primarily in the central nervous system [23], while mutation in WDR36/UTP21, a modifier protein to human primary open angle glaucoma (POAG), results in mouse embryonic lethality [24]. Mutation of Wdr36 in zebrafish doesn't produce any obvious defects in the first three days of development, while later developmental defects include small eyes and head combined with upregulation of the p53 stress-response pathway [25]. Developmental defects in other organs have also recently been reported [26]–[28].

Here, we report a novel zebrafish mutant, *fantome (fan)*, characterized by a variety of early developmental defects including eye, hindbrain, forebrain, cardiac, neurocranium, fin, and NCC derived pharyngeal arch cartilage development. NCCs in *fan* mutants fail to differentiate, and NCC precursors undergo p53 mediated cell apoptosis. *fan* mutants contain a point mutation in the zebrafish *wdr43* gene, which encodes Wdr43, the ortholog of yeast Utp5. We demonstrate that, similar to the yeast ortholog, zebrafish and human WDR43 localize to the nucleolus. We also show that the C-terminal of Wdr43, truncated in *fan* mutants, mediates its localization to nucleoli, and is both necessary and sufficient to mediate its interaction with other t-UTP subcomplex members. Interestingly, blocking WDR43 expression in HeLa cells results in nucleolar maturation defects, together with abnormal localization of other nucleolar proteins, including TCOF1. Together, our data suggest that loss function of Wdr43 results in ribosome biogenesis defects that induce the p53 signaling pathway, triggering cell death of NCC precursors. We introduce the *fan* mutant as a valuable model to provide insight into a variety of human craniofacial neurocristopathy diseases.

## Results

### Phenotype of the *fantome (fan)* mutant

**Figure 1:**
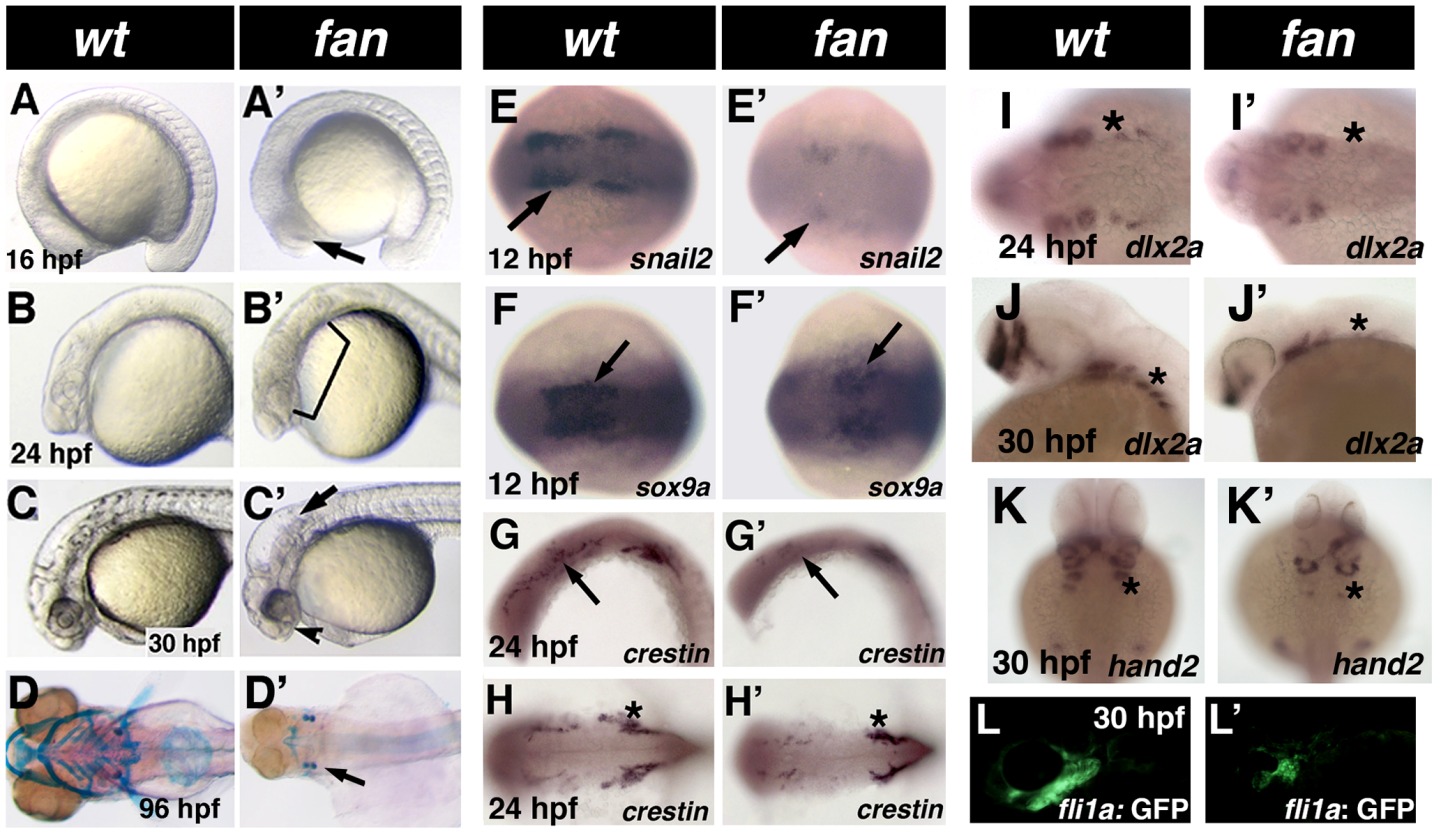
Phenotype of *fan* mutants. (A–C′) Live images of developmentally staged wild type (A–C) and *fan* mutant (A′–C′) zebrafish. Arrow in A′ indicates necrotic cells in the presumptive eye region. Bracket in B′ shows necrosis in neural and pharyngeal arch tissues. Arrow in C′ points to the distinct hydrocephaly in *fan* mutant hind brain ventricles, arrowhead indicates incomplete choroid fissure closure and craniofacial defects. (D–D′) Alcian blue stained pharyngeal arch cartilages in 4dpf wild type (D) and *fan* mutant (D′). (E–K′) Whole mount ISH images of wild type (E–K) and *fan* mutants (E′–K′) for neural crest markers at indicated developmental stage. Arrows in (E′–G′) and asterisks in (I′–K′) indicate reduced gene expression in *fan* mutant embryos. Interestingly, *fan* mutant embryos exhibit similar *crestin* expression in the trunk NCCs (asterisks in H, H′) but reduced expression in cranial NCCs. (L–L′) *Tg(fli1a:EGFP)/fan* mutants (L′) exhibit reduced GFP expression in the pharyngeal arch region as compared to wild type embryos (L).

The *fan* mutant, identified in a large scale ENU chemical mutagenesis screen conducted by the Yelick Lab [29], was notable by its distinctive lack of pharyngeal arch cartilages at 4 days post fertilization (dpf) (Fig. 1D′, arrow). Subsequent developmental analyses showed that *fan* mutants are first identifiable at 16 hpf by a distinct area of necrotic cells present in the neural ectoderm of the presumptive eye (Fig. 1A′, arrow). At 24 hpf, a larger area of necrosis was detected mainly in neural tissues (Fig. 1B′, bracket), while at 30 hpf, *fan* mutants displayed distinct hydrocephaly in hind brain ventricles (Fig. 1C′, arrow), incomplete closure of the choroid fissure, and lack of pigmented retinal epithelium in the ventral eye (Fig. 1C′, arrowhead). Preliminary whole mount in situ hybridization (WISH) analyses of NCC marker gene expression revealed that *fan* mutants exhibited reduced *snail2* and *dlx2a* expression at 12 and 24 hpf, respectively, as compared to age matched wild type sibling (Fig. 1E′, I′ vs. E, I). At 96 hpf, Alcian blue staining revealed that homozygous *fan* mutants lacked virtually all pharyngeal arch cartilages (Fig. 1D′, arrow) as compared to age matched wild type siblings (Fig. 1D). Early lethal homozygous recessive *fan* mutants die at approximately 5–6 dpf, while heterozygous *fan* embryos and adults appear normal and are viable and fertile.

In normal zebrafish development, NCCs originating from the dorsal aspect of the neural tube migrate ventrally to the pharyngeal pouches and give rise to a variety of structures including pharyngeal arch cartilages [30] [31] [32]. To more carefully characterize the pharyngeal arch phenotype observed in *fan* mutants, we used WISH to examine the developmental expression of additional NCC markers, including: *sox9a*, essential for proper morphogenesis and differentiation of pharyngeal arch cartilages [33], [34]; the pan-NCC marker *crestin*, expressed in pre-migratory and migratory NCCs [35]; *hand2*, expressed in branchial arch mesenchyme [36]; and *dlx2a*, which is expressed in migrating CNC that contribute to the pharyngeal arches [37]. We detected reduced expression of all NCC marker expression in *fan* mutants as compared to age matched wild type sibling embryos (Fig. 1F–K′). To better visualize and study NCC defects in *fan* mutants, we also created a *Tg(fli1a:EGFP)/fan* mutant reporter line, which expresses EGFP in the derivatives of the cranial neural crest until at least 7dpf, and in developing vasculature [38]. We found that *fan* mutants exhibited fewer GFP-positive NCCs, and abnormal NCC migration and pharyngeal arch formation, as compared to age matched wild type siblings (Fig. 1L–L′).

### *fan* locus encodes zebrafish Wdr43/Utp5

**Figure 2:**
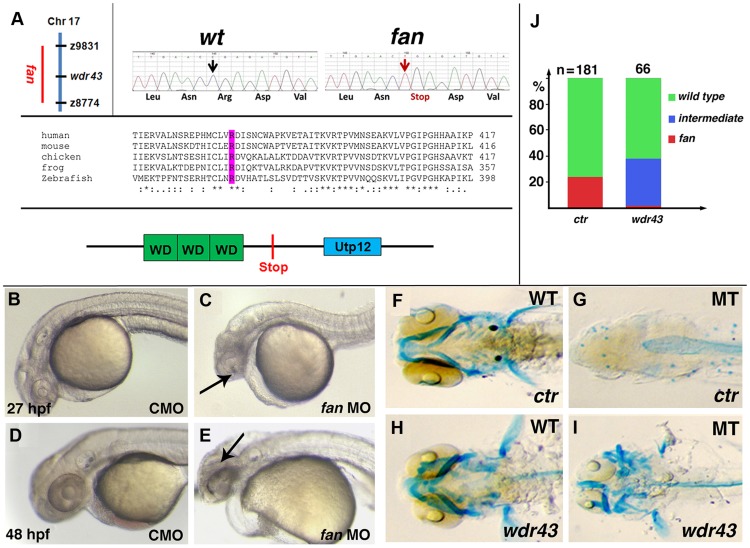
*fan* encodes zebrafish Wdr43/Utp5. (A) Left side: Chromosomal position of the *fan* locus (left). Sequencing trace data of wild type and *fan* mutant alleles, with red arrow indicating a premature stop codon in *fan* mutants (right). Comparison of the conserved nature of the amino acid mutated in *fan* zebrafish (purple) (middle). Schematic of the Wdr43 protein domains and *fan* mutant predicted premature stop codon (bottom). (B–E) Live images of zebrafish treated with control MO (CMO) or *fan* MO at indicated developmental times. (F–I) Alcian blue stained wild type (F, H) and *fan* mutant (G, I) embryos injected at the single cell stage with GFP control (*ctr*) or wild type *wdr43* mRNA. (I) Injected *wdr43* mRNA rescued *fan* mutant cartilage formation. (J) Percentage of control and *wdr43* mRNA injected *fan* mutant clutches exhibiting wild type, intermediate and *fan* mutant pharyngeal arch cartilage formation. Numbers of injected embryos scored are indicated at the top of each bar.

Using bulk segregant analyses, we determined that the *fan* locus mapped between SSLPs z8774 and z9831 on zebrafish Linkage Group (LG) 17 (Fig. 2A), to an interval of 3.93 Mb containing 11 genes. Further analyses of cDNA and genomic DNA sequences of these genes identified a cytosine to thymidine mutation at nucleotide 1066 of the *wdr43* gene in this interval, which introduced a premature stop codon at amino acid 356 in exon 9 (Arg356Stop) (Fig. 2A). This mutation was confirmed by sequencing full length *wdr43* cDNA amplified from *fan* mutant mRNA, and sequence analysis of exon 9 of the *wdr43* gene in PCR amplified genomic DNA isolated from six individual homozygous *fan* mutants. This mutation was not present in wild type sibling cDNA or genomic DNA, and was detected along with wild type sequence in heterozygous *fan* family members. Full length zebrafish Wdr43 contains 650 amino acid and is well conserved from yeast to human (Data not shown). Domain analysis of the zebrafish Wdr43 protein reveals that it is composed of three WD40 repeats and one Utp12 domain (http://pfam.sanger.ac.uk) (Fig. 2A). The truncated form of Wdr43 encoded in the *fan* mutant lack the C-term 294 aa, including the Utp12 domain (Fig. 2A).

The identified gene mutation in *fan/wdr43* mutants was confirmed using two approaches. We used single cell injections of titrated amounts of *wdr43* antisense morpholino oligomers (MOs) to test whether targeted depletion of Wdr43 in wild type embryos resulted in embryos that phenocopied the *fan* mutant. We first confirmed the functional targeting of anti-sense *wdr43* MOs by demonstrated quenching of the *wdr43-GFP* mRNA construct fluorescence *in vivo* (Fig. S1). We next injected *wdr43* MOs into clutches of *fan* mutants and wild type single cell stage embryos, which were then raised to 3–5 dpf and stained with Alcian blue to examine pharyngeal arch cartilage formation. Our results showed that wild type embryos injected *wdr43* MOs exhibited early neural tissue necrosis similar to that observed in *fan* mutants (Fig. 2C, E, arrows). When *wdr43* MOs were injected into single cell stage *fan* mutant embryos, we observed no apparent exacerbation of the *fan* mutant phenotype, suggesting that the *fan* mutation is a functional null (data not shown). WISH analyses of *wdr43* MO injected embryos revealed similar reduction in NCC marker gene expression, as observed in *fan* mutants (Figure S2).

Secondly, we performed rescues by injecting full length wild type *wdr43* mRNAs into single cell stage *fan* mutant and wild type sibling embryos. Analyses of injected embryos at 5 dpf via Alcian blue staining revealed rescue of the pharyngeal arch cartilage formation, although full rescue was not observed (Fig. 2, I versus G, F). Together, these data provide strong evidence that the identified *wdr43* gene mutation results in the *fan* mutant phenotype.

### Developmental and tissue specific expression of *wdr43*

**Figure 3:**
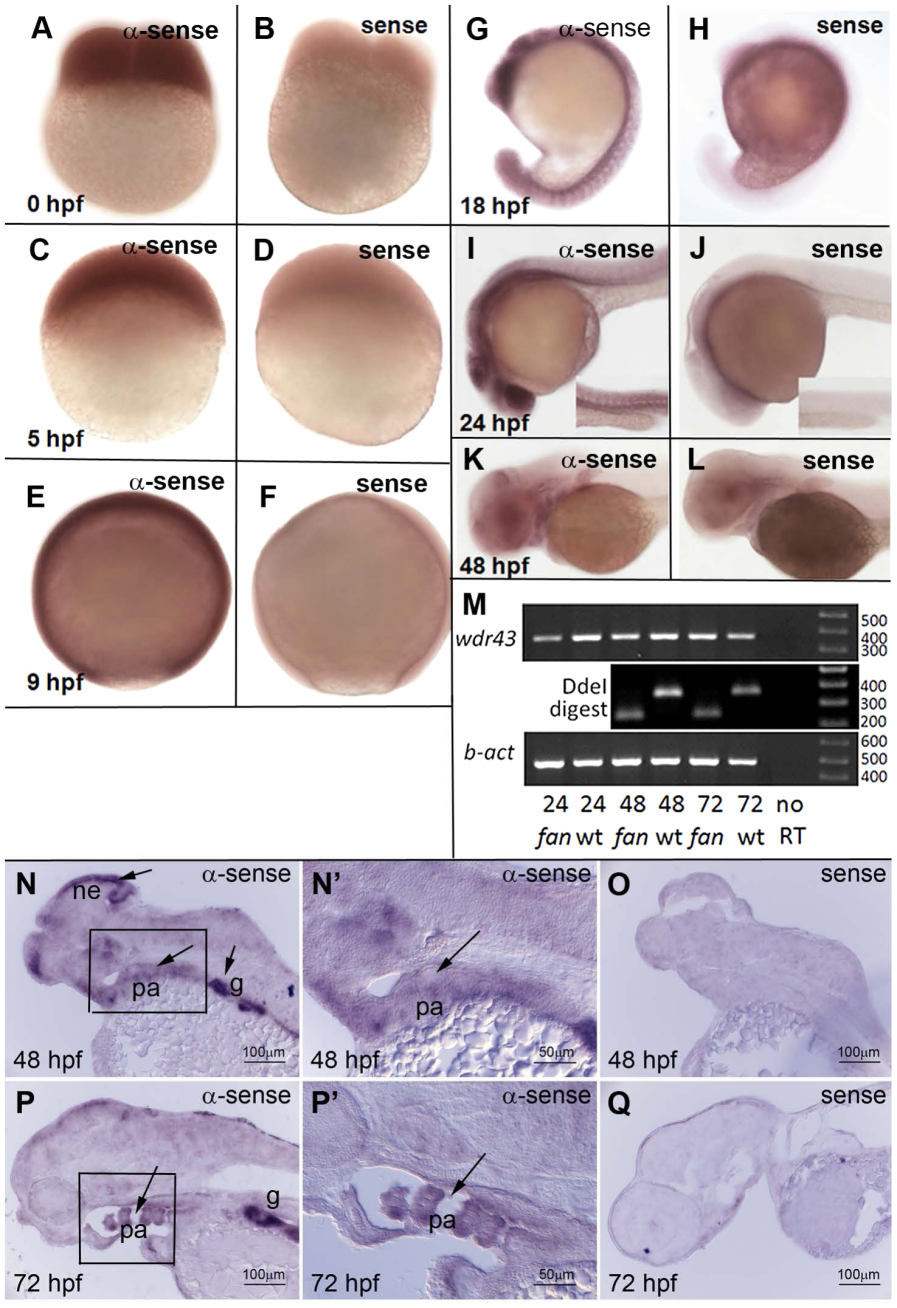
Developmental expression pattern of zebrafish *wdr43* mRNA. (A–L) Whole mount ISH images of sense and anti-sense *wdr43* probes in developmentally staged zebrafish embryos. (M) RT-PCR results of *wdr43* gene expression in wild type and *fan* mutant embryos at indicated times (hpf). *fan* mutant mRNA was detected at 48 and 72 hpf, as indicated by digestion with DdeI, a unique restriction site generated by the *fan* point mutation. (N–Q) Sectioned ISH revealed discrete *wdr43* mRNA expression in neurepithelium (ne), pharyngeal arch tissues (pa), and gut (g) (arrows). (N′, P′) Higher magnification images of boxed regions in N, P, respectively. Sense controls did not exhibit staining (O, Q).

We next examined the developmental expression pattern of *wdr43* mRNA via whole mount and sectioned in situ hybridization (ISH). *wdr43* is maternally expressed, and maintains a fairly ubiquitous expression pattern during the first 24 hours of development (Fig. 3A–J). *wdr43* expression becomes restricted to neural and pharyngeal arch tissues between 24 and 48 hpf (Fig. 3K), consistent with the pharyngeal arch defects observed in *fan* mutants. Sectioned ISH demonstrated discrete *wdr43* mRNA expression in neurepithelium and pharyngeal arches at 48 and 72 hpf (Fig. 3, N, N′, P, P′, arrows), consistent with the observed hindbrain and pharyngeal arch defects observed in *fan* mutants. Strong expression was also observed in the gut epithelium (Fig. 3, N, P). We examined the expression of *fan* mutant *wdr43* mRNA using RT-PCR analysis of developmentally staged *fan* mutant and wild type siblings followed by digestion with Dde I, a unique restriction site introduced by the *fan* allele. These analyses showed that wild type wdr43 was detectable at all stages examined, and also that mutant *wdr43* was detectable in *fan* mutants at 48 and 72 hpf, and thus was apparently not targeted for nonsense mediated decay (Fig. 3, M).

### *fantome* mutants exhibit increased apoptosis and reduced cell proliferation

**Figure 4:**
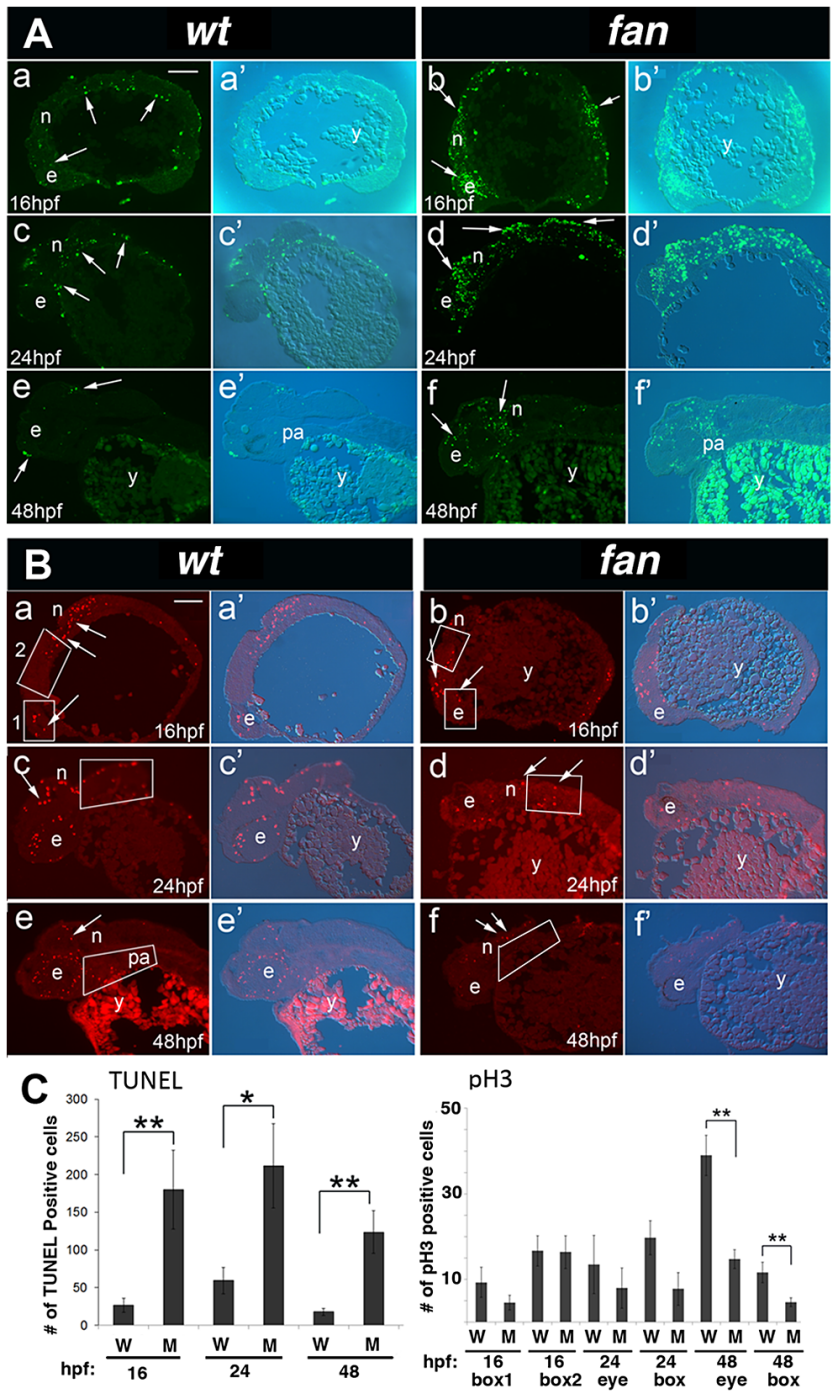
*fan* mutants exhibit increased apoptosis and reduced cell proliferation. (A) TUNEL Staining. Wild type embryos at 16 hpf (a, a′), 24 hpf (c, c′) and 48 hpf (e, e′) exhibited few apoptotic cells at all stages (arrows). In contrast, age matched *fan* mutant embryos exhibited increased levels of apoptotic cells at all stages examined (b, b′, d, d′, f, f′, arrows). Abbreviations: e, eye; n, neural tissue; pa, pharyngeal arches; y, yolk). Scale bar = 100 µm. (B) pH3 Immunofluorescent (IF) histochemistry. At 16 hpf, 24 hpf and 48 hpf, wild type embryos exhibit discrete pH3 expression in proliferating cells of the eye (e), neural tissues (n), and pharyngeal arches (pa) (a, a′, c, c′, e, e′). In contrast, age matched *fan* mutants exhibited reduced pH3 positive cell proliferation at all stages examined (b, b′, d, d′, f, f′, arrows). (Abbreviations: y, yolk). Scale bar = 100 µm. For both (A) and (B), fluorescent (a–f) and bright field plus fluorescent (a′–f′) images are shown. (C) Quantification of TUNEL and pH3 IF. For TUNEL, all apoptotic cells in each panel were counted for comparison between age matched wild type and *fan* mutant embryos. For pH3 IF, red fluorescent cells were counted in boxed areas as indicated. These results showed that *fan* mutants exhibited significantly increased apoptosis at all stages examined and significantly reduced cell proliferation at 48 hpf. At least 3 sectioned embryos were examined for each genotype at each developmental stage. Statistical analyses were performed using Student's t-test. (p = >0.01).

To better characterize tissue necrosis and cell proliferation in *fan* mutants, TUNEL assay and phosphohistone H3 (pH3) IF analyses were performed, respectively, on developmentally staged *fan* mutant and wild type sibling embryos (Fig. 4). TUNEL revealed significantly upregulated apoptosis in *fan* mutants at all developmental staged examined (Fig. 4A, arrows). In contrast, cell proliferation, indicated via pH3 antibody staining, was decreased in *fan* mutants as compared to age matched wild type siblings (Fig. 4B). Quantification of TUNEL and pH3 immunofluorescence showed significantly increased apoptosis in *fan* mutants as compared to age matched wild type siblings at all stages examined, and significantly decreased cell proliferation at 48 hpf (Fig. 4C). Together, these results are consistent with the observed lack of NCC derived pharyngeal arch tissues in *fan* mutants.

### Nucleolar localization of wild type and *fan* mutant Wdr43

**Figure 5:**
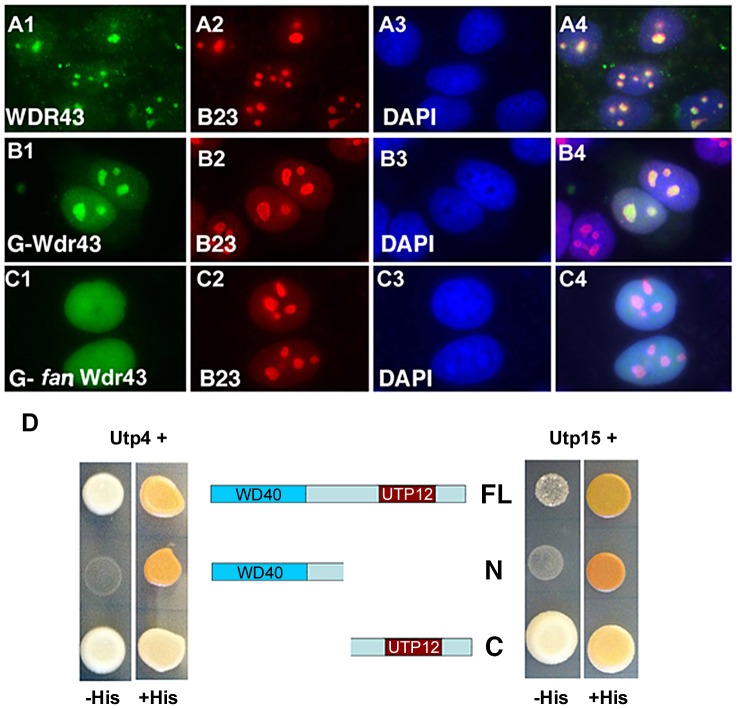
Subcellular localization and Y2H analyses of wild type and *fan* mutant Wdr43. (A1–A4) IF images of HeLa cells immunostained with anti-WDR43 (green) and anti-B23 (red) antibodies followed by DAPI stain (blue) to visualize nuclear DNA. IF images of Hela cells transfected with EGFP tagged zebrafish wild type Wdr43 (B1–B4) or *fan* mutant Wdr43 (C1–C4). Anti-GFP antibody was used to increase the fluorescent signal of EGFP-tagged wild type and *fan* mutant Wdr43 expressed proteins. Stained cells were counterstained with anti-B23 (red) and DAPI (blue). (D) Y2H analysis of zebrafish full length (FL) Wdr43, truncated *fan* mutant Wdr43 (N), and Wdr43 C-terminal domain (C) interactions with zebrafish Utp4 and Utp15 proteins.

To better understand the molecular nature of mutant Wdr43 protein, we next examined the subcellular localization of wild type and *fan* mutant zebrafish Wdr43 in human cells. First, we performed immunofluorescence analyses of cultured HeLa cells using the anti-human WDR43 antibody and demonstrated that endogenous human WDR43 localized to nucleoli, as shown by co-localization with the nucleolar marker protein, B23 (Fig. 5, A1–A4). Next, we generated N-terminal EGFP-tagged wild type and truncated *fan* mutant zebrafish *wdr43* constructs driven by the CMV promoter, which we transfected into cultured HeLa cells, and then visualized chimeric fusion protein using anti-GFP antibody. The EGFP-tagged wild type Wdr43 protein showed perfect overlapping expression pattern with B23 (Fig. 5, B1–B4), consistent with the previously characterized nucleolar localization of the yeast ortholog for Wdr43, Utp5 [39]. In contrast to the full length EGFP-Wdr43 protein, EGFP-tagged *fan* mutant Wdr43 (amino acids 1–364) lost its exclusive nucleolar localization, and rather exhibited expression throughout the nucleus (Fig. 5, C1–C4). To correlate these *in vitro* results to *in vivo* expression studies in zebrafish, the same EGFP-tagged zebrafish wild type and *fan* mutant *wdr43* constructs were injected into single cell stage zebrafish, which were then analyzed via confocal microscopy (Fig. S3). These analyses also showed that while full length Wdr43 co-localized with mCherry-B23 to nucleoli (Fig. S3, A–C), truncated *fan* mutant Wdr43 remained dispersed throughout the nucleus (Fig. S3, D–F).

### Protein-protein interactions between wild type, *fan* mutant Wdr43 and t-Utp subcomplex proteins

Utp5, the yeast ortholog of Wdr43, has been shown to function in the yeast t-Utp subcomplex, which mediates both pre-ribosomal RNA (rRNA) transcription and processing [16] [17]. Previously, it has been shown that yeast Utp5 interacts with Utp4 and Utp15 (Freed & Baserga 2010) [40]. We used yeast two-hybrid analyses (Y2H) to examine the protein-protein interactions between yeast and zebrafish wild type and *fan* mutant Wdr43 with other t-Utp complex proteins. We found that both yeast and zebrafish full length Wdr43 interacted with Utp4 and Utp15 (Fig. 5D and Fig. S4), consistent with previously published yeast Utp5/Wdr43 binding studies [17]. We also determined that the C-terminal portion of Wdr43 protein is both necessary and sufficient to mediate this interaction. Zebrafish and yeast truncated *fan* mutant Wdr43 did not bind to either Utp4 or Utp15, while the C-terminal fragment of Wdr43 alone was able to bind to both Utp4 and Utp15 (Fig. 5D zebrafish and Fig. S4 yeast). Together, these data revealed the conserved interaction of yeast and zebrafish full length Wdr43 proteins with Utp4 and Utp15, and also suggest that the C-terminal portion of Wdr43, which contains the Utp12 domain, is required for protein interaction of Wdr43 with other t-UTP subcomplex member proteins.

### Wdr43 is required for optimal pre-rRNA transcription and proper ribosomal protein sub-nucleolar localization

**Figure 6:**
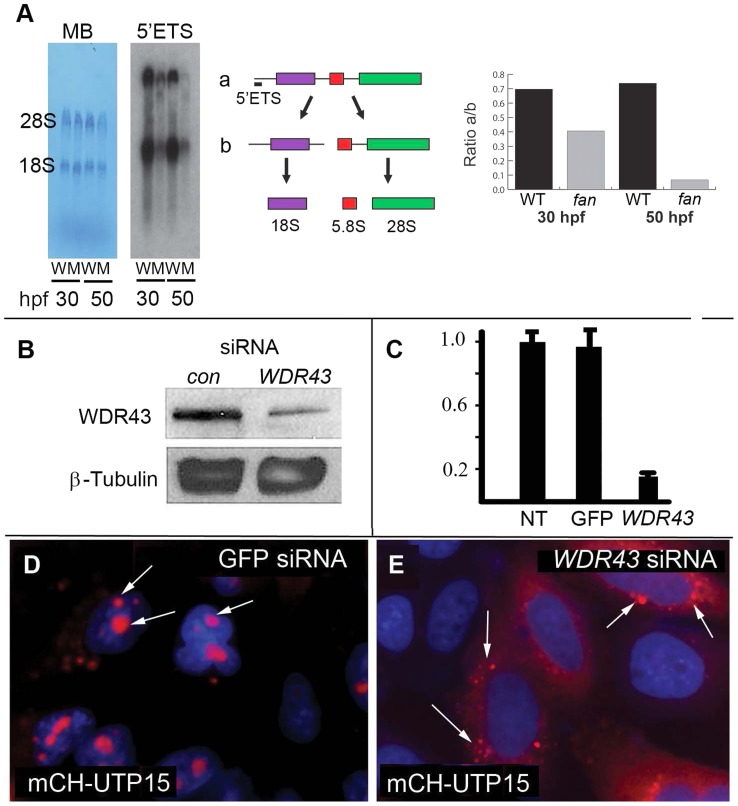
Ribosome biogenesis defects in *fan* mutant zebrafish. (A) Northern blot analysis of rRNA isolated from 30 and 50 hpf wild type (W) and *fan* mutants (M) using an oligonucleotide probe against the 5′ETS region of zebrafish pre-rRNA. Pre-rRNAs (a) and (b) are indicated. Methylene blue (MB) staining of the mature 28S and 18S rRNAs was carried out as a loading control. Quantitation of the ration of a/b was performed using Image J. (B) Western blot of whole-cell extracts treated with *WDR43* or EGFP control siRNA as indicated. (C) qRT-PCR analyses of human *WDR43* gene expression level in non-treated (NT), GFP or *WDR43* siRNA treated HeLa cells normalized to ß -actin. (D, E) Subcellular localization of mCherry-tagged Utp15 (red) in control GFP (D) and *WDR43* (E) siRNA treated HeLa cells counter stained with DAPI (blue).

Based on our results, and those of previously published reports, we anticipated that Wdr43 would play an important role in ribosome biogenesis. We therefore investigated pre-rRNA synthesis and processing in 30 hpf and 50 hpf *fan* mutant and wild type sibling embryos via Northern blot analysis, using a probe specific for the 5′ external transcribed spacer (5′ETS) region of the pre-rRNA at the start site of transcription (Fig. 6A). These analyses showed reduced levels of the primary transcript (labeled a) in *fan* mutants (M) at both 30hpf and 50 hpf as compared to that of wild type (W) siblings, consistent with a defect in pre-rRNA transcription in *fan* mutants. Quantification of the ratio of full length primary transcript (a) to the processed pre-18S rRNA (b) showed reduced pre-rRNA in *fan* mutants relative to age matched wild type siblings. These results are consistent with previously published results showing that siRNA knockdown of human UTP5 resulted in defects in pre-rRNA transcription and processing [19], and suggest conserved functions for vertebrate Wdr43 and yeast Utp5/Wdr43 in pre-rRNA transcription [17].

Having defined an important role for zebrafish Wdr43 in ribosome biogenesis, we further examined its function in cultured human HeLa cells, which we transfected with human *WDR43* small interfering RNA (siRNA) (Sigma MISSION esiRNA) to silence WDR43 expression. GFP esiRNA was used as a negative control for these studies. Analysis of WDR43 protein and mRNA expression in siRNA treated cells using both Western blot (Fig. 6B) and qRT-PCR (Fig. 6C) analyses, respectively, confirmed that endogenous WDR43 expression was significantly reduced with *WDR43* siRNA treatment. We next examined how WDR43 depletion affected the localization of another t-UTP complex Wdr43 interacting protein, UTP15. Due to the lack of available antibody for UTP15, we transfected N-terminal mCherry tagged zebrafish UTP15 into HeLa cells, and monitored its localization via fluorescent confocal microscopy. Consistent with a role for UTP15 in pre-rRNA processing, we found that mCherry tagged UTP15 localized to nucleoli in control GFP siRNA treated cells (Fig. 6D). In contrast, in HeLa cells depleted of WDR43 using *WDR43* siRNA, mCherry-tagged UTP15 did not localize to nucleoli, but rather appeared exhibited a perinuclear expression pattern (Fig. 6E). These results indicate that WDR43 is required for entry into the nucleus, as well as for proper nucleolar localization of UTP15.

**Figure 7:**
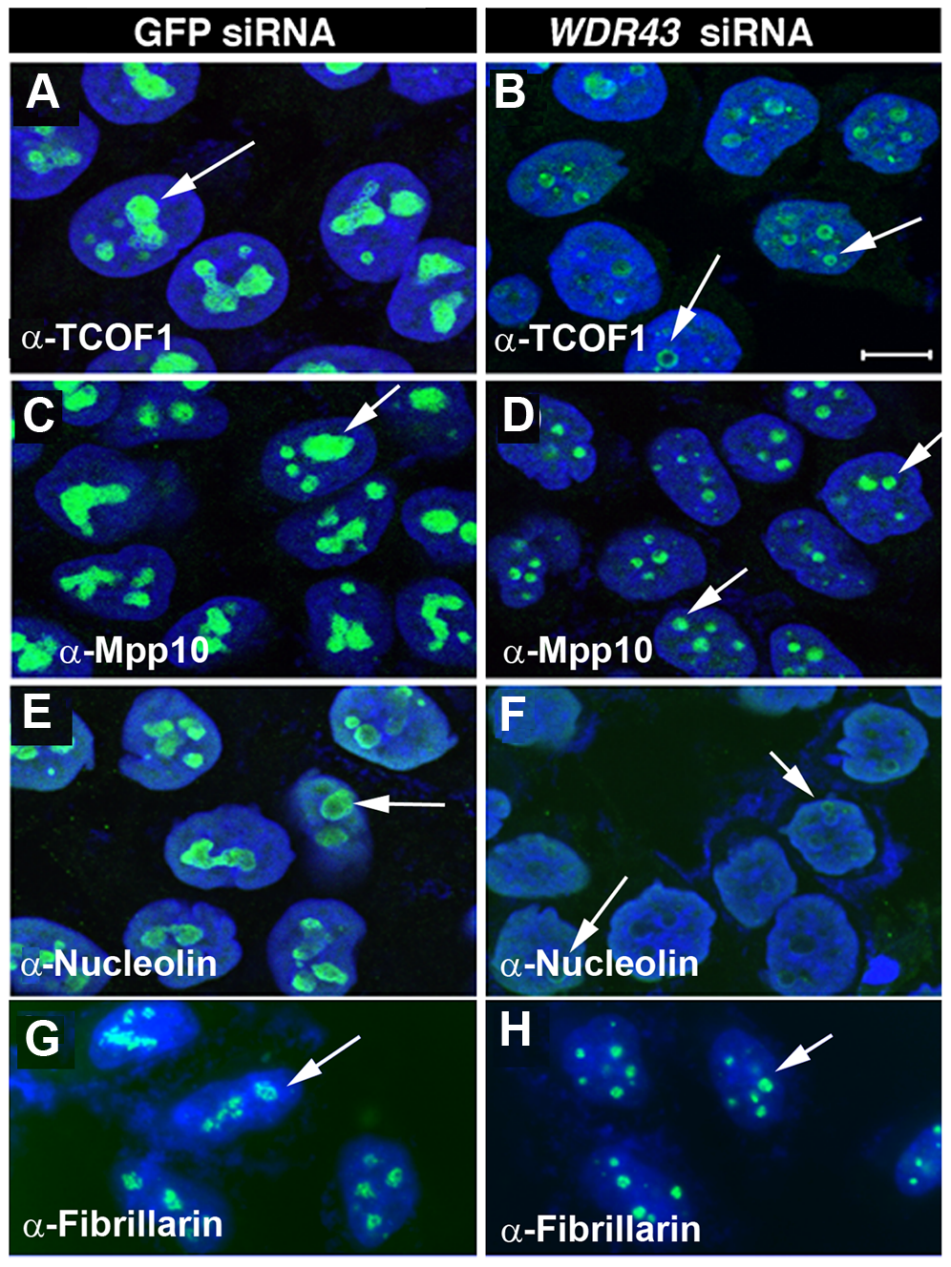
Subcellular localization of nucleolar proteins in WDR43 depleted HeLa cells. IF analysis of nucleolar protein localization in control siRNA (A, C, E, G) and *WDR43* (B, D, F, H) siRNA treated HeLa cells. WDR43 depleted cells exhibited mislocalized expression of TCOF1 (B), Mpp10 (D), Nucleolin (F) and Fibrillarin (H) (arrows), as compared to their respective control GFP siRNA treated cells (see arrows). Scale bar = 10 µm.

**Figure 8:**
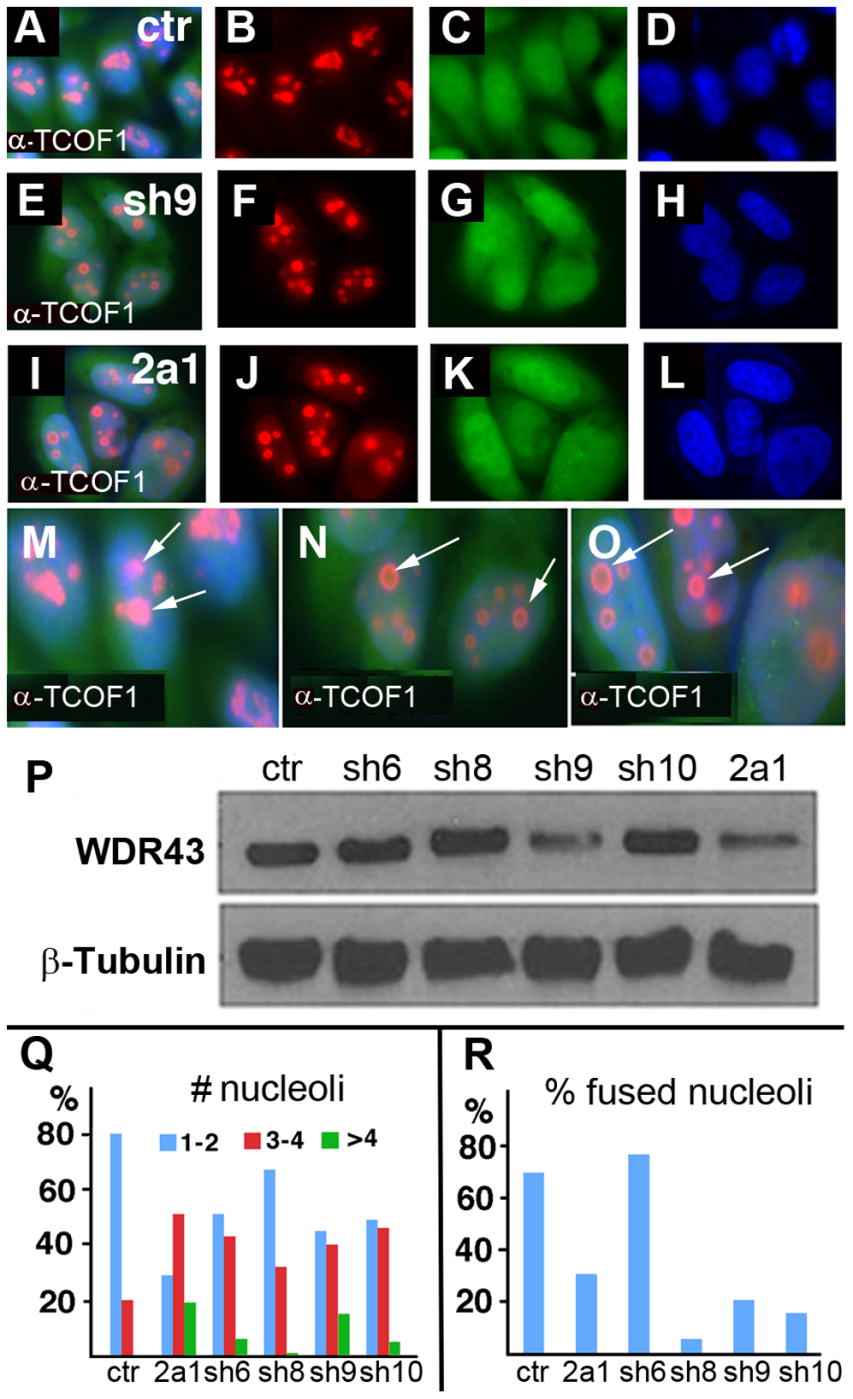
TCOF1 expression in stable *WDR43* shRNA cell lines. (A–O) IF analysis of TCOF1 (red) localization in control shRNA (A–D, M) and human *WDR43* shRNA (E–L, N, O) stable HeLa cell lines. All cells were positive for shRNA expression (green) and DAPI stained nuclei (blue). (P) Western blot analyses of WDR43 expression in stable *WDR43* shRNA cell lines. (Q–R) Quantification of TCOF1 positive nucleoli per cell (Q), and percent cells with fused TCOF1 positive nucleoli (R), in control and *WDR43* shRNA stable lines. The number of nucleoli was increased (Q), and the percentage of fused nucleoli was reduced (R) in WDR43 depleted cells, as compared to control cells. Sample numbers: Panel Q - ctr 290, 2a1 136, sh6 195, sh8 172, sh9 172, sh10 108; Panel R – ctr 268, 2a1 115, sh6 181, sh8 163, sh9 171, sh10 108.

It was intriguing to us that many of the observed phenotypes observed in *fan* mutant zebrafish have also been reported in humans (and mice) with mutations in *TCOF1/Treacle*, the gene commonly mutated in Treacher-Collins Syndrome (TCS). TCS results in aberrant NCC specification and differentiation, craniofacial dysmorphologies, increased cell apoptosis, and upregulated p53 signaling [10], [11]. Based on these common characteristics, we investigated whether the localization of TCOF1 and other nucleolar proteins was affected in human HeLa cells depleted of WDR43 protein. For reference, we examined the expression of the nucleolar proteins Mpp10, Nucleolin and Fibrillarin, which have been associated with distinct nucleolar functions. Mpp10 is normally found in the dense fibrillar component (DFC) and in the boundary between the DFC and the fibrillar center (FC), sites of rDNA transcription and pre-rRNA splicing and modification by snoRNPs, while Fibrillarin is normally found in association with snoRNAs throughout the DFC [41]. Nucleolin/C23 is normally localized to the outer layer of nucleoli with fainter expression at the center [42]. Our investigation of the expression of these nucleolar proteins, and TCOF1, in control and *WDR43* siRNA treated HeLa cells (Figure 7) showed that TCOF1 exhibited a reduced and perinucleolar expression pattern in WDR43 depleted cells (Fig. 7 B vs. A, arrows). Mpp10 expression appeared reduced, but was expressed throughout the smaller nucleoli (Fig. 7 B vs. D, arrows). Nucleolin, although barely detectable in *WDR43* siRNA expressing cell lines as compared to control GFP siRNA treated cells, was also expressed in a perinucleolar fashion, similar to that of TCOF1 (Fig. 7 F vs. E, arrows). In contrast, Fibrillarin expression appeared relatively less affected in *WDR43* depleted cells, and was detected throughout the smaller nucleoli (Fig. 7H vs. G, arrows). These results are indicative of disrupted nucleolar organization and rRNA transcription, consistent with the observed defects in pre-rRNA transcription observed in *fan* mutants. To confirm and more reliably study these observations, we next examined TCOF1 localization in stable HeLa cell lines expressing stable short hairpin RNAs (shRNAs) for GFP control and *WDR43* (Fig. 8). We first tested five shRNAs against *WDR43* gene and found that all of them showed somewhat reduced *WDR43* expression using qRT-PCR analysis (Fig. S5). Western blot analyses showed that two of the shRNA *WDR43* cell lines, sh9 and 2a1, exhibited the most efficient inhibition of protein levels. As observed in *WDR43* siRNA treated cell lines, stable *WDR43* shRNA expressing cell lines sh9 and 2a1 exhibited mislocalized TCOF1 expression at the periphery of nucleoli, as compared to control shRNA stable cell lines (Fig. 8, E, I, N, O vs. A, M, arrows). Together, these results suggest that WDR43 expression is required for proper nucleolar organization and the subnucleolar localization of a variety of nucleolar proteins including TCOF1, and for optimal pre-rRNA transcription.

### WDR43 is required for proper nucleolar morphology, maturation and assembly

We also found that WDR43 depletion had an effect on nucleolar size and shape. WDR43 depleted cells had larger numbers of mini nucleoli as compared to control cells, which exhibited fewer numbers of larger sized, mature nucleoli. To quantitate this observation, we monitored nucleolar number and size in control and WDR43 depleted cultured HeLa cells immunostained for TCOF1 (Fig. 8). We found that in normal and control shRNA cultured HeLa cells, nucleoli reassembled after mitosis, with several small nucleoli fusing into ∼1–4 larger, mature nucleoli per HeLa cell. In contrast, TCOF1 expressing nucleoli failed to fuse together in *WDR43* shRNA expressing HeLa cells, but rather remained as small unfused mini nucleoli, or “nucleolar caps”, which also appeared spherical shape as compared to the irregular shaped nucleoli present in control HeLa cell cultures (Fig. 8 F, J vs. B). We also found that the average number of nucleoli was increased, and the number of fused nucleoli was reduced, in *WDR43* shRNA expressing HeLa cells as compared to control shRNA treated HeLa cells, as shown using the nucleolar marker B23 (Fig. 8, F, J vs. B). Quantification of these results revealed that WDR43 depleted cells exhibited increased numbers of smaller nuclei as compared to control cells (Fig. 8, Q, R). Together, these results demonstrate that depletion of WDR43, an essential ribosome biogenesis factor, affects nucleolar maturation and assembly.

### *fan* mutants exhibit upregulated p53 signaling

**Figure 9:**
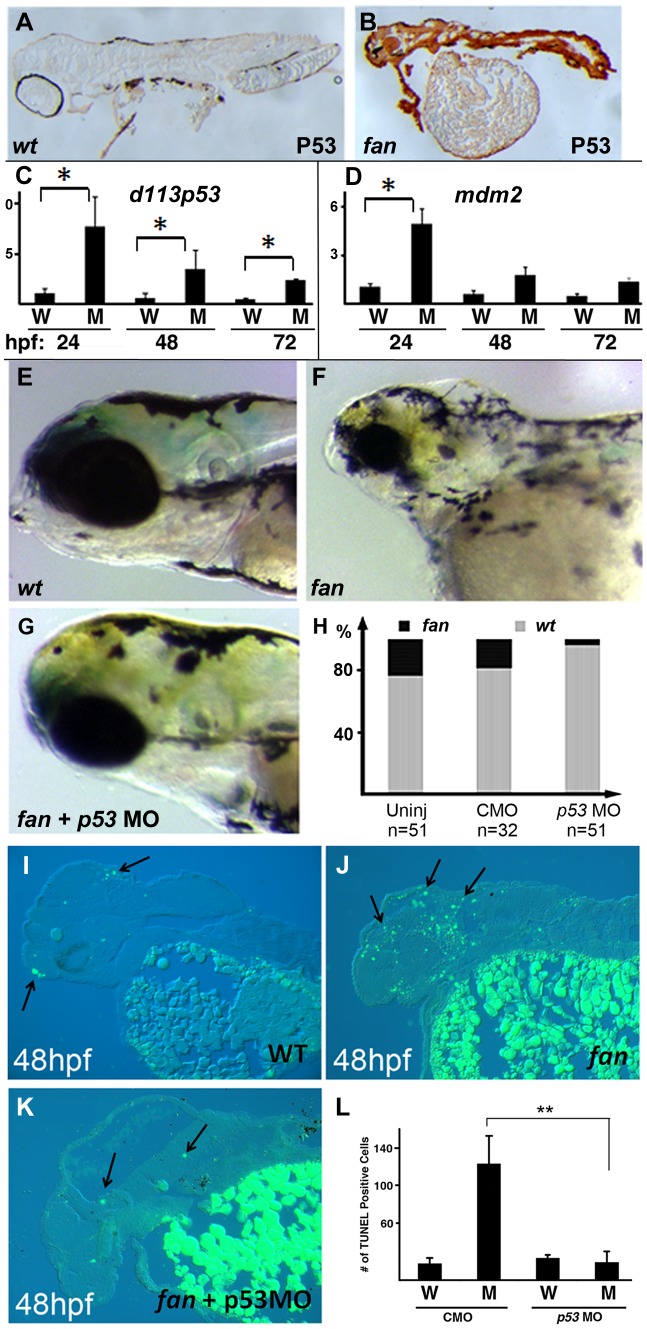
Inhibition of p53 signaling can partially rescue *fan* mutant craniofacial defects. (A–B) Whole mount IHC analysis of p53 expression in wild type (A) and *fan* mutants (B). (C, D) qRT-PCR analysis of *d113p53* (C) and *mdm2* (D) gene in wild type and *fan* mutants at 24, 48 and 72 hpf. (E–G) Bright field images of live 3 dpf wild type (E), *fan* mutant (F), and *fan* mutant injected with p53MO (G). (H) Percent zebrafish exhibiting normal eye development in uninjected (Uninj), control MO (CMO), and p53MO injected embryos. (I–K) TUNEL stained and sectioned wild type (WT) (I), *fan* mutant (J), and *fan* mutant injected with p53 MO (K). Arrows indicate apoptotic cells. (L) Statistical analyses revealed significantly reduced apoptosis in *fan* mutants injected with p53MO. (P value<0.01).

Ribosome biogenesis defects, such as those observed in *fan* mutants, have been reported to be associated with upregulation of the p53 signaling pathway, and cellular apoptosis [11], [25] [43] [44] [45] [46]. Based on the increased apoptosis observed in *fan* mutants, we next examined p53 signaling pathway gene expression in developing *fan* mutant and wild type sibling embryos. Immunohistochemical analysis using the zebrafish p53 antibody (kind gift of David Lane) [47] revealed high levels of expression of p53 in *fan* mutants, while p53 was virtually undetectable in wild type sibling embryos at 5 dpf (Fig. 9, B vs. A). Next, we used qRT-PCR analyses to show that the expression of p53 downstream target genes, including the N-terminal truncated p53 isoform *delta113p53*, *mdm2*, and *cyclin G1* were all upregulated in 24, 48 and 72 hpf *fan* mutants as compared to wild type sibling controls (Fig. 9, C, D and data not shown). We next tested whether knockdown of p53 signaling via injection of p53 anti-sense MOs could rescue the *fan* mutant phenotype. Similar to previous reports in the Treacher-Collins mouse model [11], we found that the *fan* mutant pharyngeal, neural and eye defects were largely rescued in p53 MO injected *fan* mutants (Fig. 9, G vs. F, and H), and NCC marker gene expression was also rescued in *fan* mutants (Fig. S2). TUNEL analyses revealed rescue of apoptosis in p53MO injected *fan* mutants (Fig. 9. K vs. J, I), indicating that increased apoptosis observed in *fan* mutants was mediated via upregulated p53 signaling pathways.

## Discussion

Proper ribosome biogenesis is required for the production of functional ribosomes, the primary site of protein synthesis. Most if not all ribosomal proteins (RPs) are thought to be essential for ribosome biogenesis and cell survival. It is therefore surprising that ribosome biogenesis defects caused by mutations in certain RPs can lead to variable and seemingly tissue-specific defects in vertebrate development. For example, mutations in several RPs are associated with human congenital hypoplastic Diamond-Blackfan anemia (DBA), and similar DBA phenotypes were observed when DBA associated RP mutations were expressed in zebrafish [48]–[51]. In addition, several reports on zebrafish ribosome biogenesis protein mutants describe a variety of diverse phenotypes, ranging from tumors, to central nervous system degeneration, to organogenesis defects [23], [25], [52], [53].

Here we present data showing that defects in the zebrafish ribosome biogenesis protein Wdr43 result in early developmental defects in a variety of tissues, including neural, eye and heart and pharyngeal arches, while later developmental defects appear fairly localized to NCC derived pharyngeal arch cartilages. These observations raise the question of how a defect in what is thought to be a universally required ribosomal biogenesis protein, Wdr43, can result in a rather specific craniofacial tissue-specific phenotype? One possibility is that there are tissue specific, developmental requirements for ribosome biogenesis proteins. For example, certain ribosome biogenesis factors may have tissue specific, developmental expression patterns. In fact, we show here that the expression of zebrafish *wdr43* mRNA becomes localized to neural and pharyngeal arch tissues starting at ∼24 hpf, which is consistent with the observed *fan/wdr43* mutant phenotype. It is possible that additional ribosomal proteins that regulate cell cycle or apoptosis may similarly exhibit tissue specific expression patterns. Another theory is that ribosome biogenesis defects and subsequent anticipated reduced protein translation efficiency will most significantly affect those tissues exhibiting a high demand for protein synthesis. This may include NCC and erythropoiesis progenitor cells, although recent data does not find evidence for increased translation in NCC [21]. Finally, decreased efficiency of cellular translation machinery may affect a wide and varied spectrum of translation products in different cell types due to mRNA competition for timely translation, which could result in diverse readouts in different cell types.

The craniofacial phenotype exhibited by zebrafish *fan* mutants resembles the craniofacial malformations observed in individuals with Treacher-Collins Syndrome. The fact that NCC specification and differentiation are similarly affected by mutations in nucleolar proteins – TCOF1/Treacle and a common subunit of RNA polymerases I and III in Treacher-Collins Syndrome [54] [55], and Wdr43 in *fan* mutants - leads to the intriguing question of why NCCs may be more sensitive to ribosome biogenesis defects as compared with other tissues. Based on our data presented here and on the published reports of others, we hypothesize that high protein translation levels must be maintained by progenitor and differentiating NCCs in order to support their extensive cell proliferation, migration and differentiation. In Treacher-Collins Syndrome, *TCOF1* mutant induced defects in ribosome biogenesis are characterized by stimulation of the nucleolar stress response, which in turn activates the p53 apoptosis pathway, resulting in the depletion of the neural crest precursor pool [10]. We observe a similar upregulation of p53 signaling and depletion of NCCs in *fan* mutants. Although beyond the scope of the present study, it will be interesting in future studies to compare pre-rRNA transcription, ribosome biogenesis and protein translation efficiency in developmentally staged NCC versus non-NCC populations harvested from *fan* mutant and wild type siblings.

Our Northern blot results indicated that pre-rRNA levels are significantly decreased in developmentally staged zebrafish *fan/wdr43/utp5* mutants, consistent with the previously characterized role for yeast Utp5 in pre-rRNA transcription [16], [19]. We suggest that a variety of nucleosomal proteins are required for optimal pre-rRNA transcription. Novel findings from this report include the fact that blocking WDR43 function in HeLa cells resulted in the distinct mislocalization of nucleolar proteins including UTP15, Mpp10, nucleolin and to a lesser extent fibrillarin, suggesting that Wrd43/UTP5 is required for proper subnucleolar organization and function. Interestingly, TCOF1 also mislocalized to the outer periphery of nucleoli, rather than exhibiting its normal distribution throughout the nucleolus, suggesting that WDR43 may also be required for proper TCOF1 subnucleolar localization and function. Although we have not detected direct binding between TCOF1 and WDR43/UTP5 using Y2H, we have detected interactions between WDR43/UTP5 and other rDNA transcription component proteins (data not shown). Together, these results suggest roles for Wdr43/UTP5 in ribosomal protein sub-nucleolar localization and function of other ribosome biogenesis factors, and raise the intriguing possibility that manipulation of WDR43 expression could be used to correct the localization and improve the function of TCOF1 in Treacher-Collins Syndrome patients.

Nucleolar mis-localization phenotypes have also been observed in HeLa cells treated with actinomycin D, an inhibitor of RNA Pol I [56], which is a TCOF1/Treacle interacting protein [57]. It is possible that WDR43 may also function together with TCOF1 and Nopp140 to recruit proteins to the nucleolar organizer regions (NORs) and the upstream binding factor (UBF), an RNA PolI transactivator [19]. Wdr43 could also mediate rRNA transcription by binding to rDNA and UBF directly, as shown by other Utps [58]. These functions for Wdr43 remain to be elucidated.

We also used both siRNA and shRNA *WDR43* silencing methods to confirm the function of WDR43 in nucleolar fusion in cultured HeLa cells. At the present time, mechanisms regulating nucleolar fusion remain poorly understood. It has been shown that after mitosis, multiple small nucleoli form around transcriptionally active NORs, and as cells progress through the cycle, these small nucleoli fuse to form larger nucleoli [59] [60]. Although the mechanism of WDR43 function in nucleolar fusion is not clear, preventing nucleolar fusion may not be common to all ribosome biogenesis protein mutations based on the fact that inhibition of NOL11 resulted in the formation of one large (not small) nucleolus [20]. One possible explanation is that WDR43 depletion may result in structural changes to rDNA, which in turn could interfere with nucleolar fusion [61]. Such a proposed function for WDR43 may be dependent or independent of its function in the t-Utp complex. A recent study using *Xenopus* oocytes showed that nucleoli exhibit fluid dynamics similar to that of liquid droplets, and that nucleolar fusion requires dynamic exchange between nucleoli and the nucleoplasm [62]. In future studies, it will be interesting to determine whether WDR43 is also involved in this process.

Recent reports emphasize the apparent tissue specific functions for ribosomal proteins previously thought to exhibit functions in all cells and tissues. The results presented here suggest previously unrecognized roles for Wdr43/UTP5 in craniofacial development. The fact that Wdr43/UTP5 is needed for proper formation of nucleoli and for sub-nucleolar organization and function indicates important roles for Wdr43 as a key participant in ribosome biogenesis. As such, the zebrafish mutant *fantome* provides a valuable vertebrate developmental model and tool to continue in depth functional studies of RPs and ribosome biogenesis factor proteins in NCC differentiation, including the identification of effective tools for reducing the incidence of craniofacial birth defects.

## Materials and Methods

### Zebrafish husbandry

AB and WIK *fantome/wdr43* mutant and wild type zebrafish were raised in the Tufts Zebrafish Facility at 28.5°C and developmentally staged as previously described (Westerfield, M., 1995). For whole mount in situ hybridization analyses, pigmentation was inhibited by treating embryos with 1-phenyl-2-thiourea (PTU) at a final concentration of 0.2 mM as previously described (The Zebrafish Book, U. Oregon Press).

### Ethics statement

All experimental procedures on zebrafish embryos and larvae were approved by the Tufts University Institutional Animal Care and Use Committee (IACUC) and Ethics Committee.

### Positional cloning of the *fantome* mutant locus

The *fan* mutant was identified in a large-scale ENU-mutagenesis screen conducted by the Yelick Laboratory [29]. Genetic mapping strains were created by crossing identified heterozygous *fan* mutants to polymorphic WIK wild type zebrafish. Embryos were collected from pairwise matings of mapping strain *fan/WIK* heterozygotes, and scored at 48 hpf for *fan* specific phenotypes. Genomic DNA was extracted from individual *fan* mutant and wild type embryos, and bulk segregant analyses were performed using primers designed to amplify SSLP markers from the Massachusetts General Hospital Zebrafish Server website (http://zebrafish.mgh.harvard.edu). The *fan* mutation mapped to zebrafish linkage group 17 (LG17), to a region spanning 11 genes. Nucleotide sequence analyses of all 11 genes identified a premature stop codon in the *wdr43* gene of all *fan* mutant embryos that was not present in wild type siblings.

### In situ hybridization

Whole-mount and sectioned in situ hybridizations were performed as previously described (Thisse et al., 1993), using a probe generated via PCR using the following primers (*wdr43*-forward: 5′- CAGTGCAACAAAAGTTGGTGA-3′; *wdr43*-reverse: 5′- AAAGTTCTGGTTGGCTGCA-3′). All other probes were obtained from zfin.org. Embryos were analysed using Zeiss Axiophot and M2Bio microscopes, and imaged using Zeiss Axiophot Imager digital camera (Munich, Germany). Digital images were processed using Adobe Photoshop software.

### Targeted protein depletion of Wdr43/UTP5 using anti-sense *wdr43* morpholino oligomer (MO) injections

Antisense morpholino oligonucleotides (MOs) targeted to the initiation of translation codon of *wdr43* mRNA (5′TCCGTCCGCCGCCATCTTACCGTTC3′) were injected into the yolk of 1 cell stage wild type or fan mutant embryos. 2 nL of MO at a concentration of 10 ng/µL was used to knockdown *wdr43* translation.

### Quantitative reverse transcriptase polymerase chain reaction (qRT-PCR)

Total RNA was extracted from 20 wild type and 20 *fan* mutant embryos at 24, 48 and 72 hpf, or from HeLa cells 48 hours after transfection using RNeasy Plus Kit (Qiagen, Valencia, CA). DNA was removed using the DNA-*free* DNase Treatment & Removal Kit (Ambion,Life Technologies, Grand Island, NY) to remove genomic DNA contamination. cDNA was synthesized using a SuperScript III First-Strand Synthesis System (Invitrogen, Life Technologies, Grand Island, NY) with random primers. Gene expression was quantified by qRT-PCR using QuantiTect SYBR Green PCR Master Mix (Qiagen, Valencia, CA) and real-time cycler Mx3000P (Stratagene, Agilent Technologies, Santa Clara, CA). Primers for zebrafish *p53 isoforms*, *mdm2*, and *cyclinG1* were used as described [63]. The following primers were used to amplify the human *WDR43* gene: Forward: CCTTCCGCGCACCTCAGTGGTAC; Reverse: AACTGGCGTTGCATGTCCTGTGA. Primers for β-actin, used to normalize the expression levels, were as described [64].

### Yeast two-hybrid analysis

Yeast two-hybrid assays for interaction between yeast and zebrafish Utp proteins were performed as previously described (Freed and Baserga, 2010). Briefly, yeast *utp5* cDNA encoding full length, N-terminal (1–343aa) and C-terminal (344–643aa) proteins were cloned into the pGADT7 prey vector. Additional yeast UTP genes of the t-Utp subcomplex (Utp8, Utp9, Utp10, Utp15 and Utp17) cloned into the bait vector were as previously described (Freed and Baserga 2010). Both bait and prey vectors were transformed into AH109 yeast strain and interactions were identified based on the ability of transformants to grow on AHTL dropout medium after 3–5 days of incubation at 30°C. To test the interaction between zebrafish Wdr43/Utp5, Utp4 and Utp15, full length zebrafish *utp4* and *utp15* cDNAs were purchased (Openbiosystems, Lafayette, CO) and cloned into pGADT7 prey vector. Zebrafish *wdr43* cDNAs encoding full length, truncated *fan* mutant Wdr43 (1–356aa) and C-terminal portion of Wdr43 (357–650aa) proteins were cloned into the pGABT7 bait vector. Interactions were tested by growth in triple dropout medium after 3–5 days of incubation at 30°C. The Y2H studies of zebrafish Wdr43 protein interactions were tested in both bait and prey constructs.

### Wdr43 subcellular localization

To determine the subcellular localization of wild type and mutant Wdr43 proteins, *GFP* cDNA was cloned onto the N-terminal end of full length or *fan* mutant zebrafish *wdr43* cDNA under the direction of the CMV promoter, using multi-site Gateway reactions [65]. These constructs were then transfected into HeLa cells using Lipofectamine 2000 reagent (Invitrogen, Life Technologies, Grand Island, NY). After 36 hours of growth at 37°C, transfected cells were fixed with 4% PFA, and subjected to standard immunofluorescence (IF) analyses using the anti-B23 antibody (1∶200, Santa Cruz Biotechnology, Inc., Santa Cruz, CA). The rabbit polyclonal anti-WDR43 antibody (1∶100, Abcam, Cambridge, MA) was used to detect the endogenous WDR43. To check the expression in zebrafish embryos, the same constructs were injected into single cell zebrafish embryos, which were harvested at 24hpf, and analyzed for GFP expression using a Leica TCS SP2 confocal microscope.

### RNA interference

For siRNA experiments, HeLa cells were transfected with WDR43 MISSION esiRNA (Sigma, EHU004691) using Lipofectamine 2000 reagent (Invitrogen, Life Technologies, Grand Island, NY). GFP esiRNA (Sigma, EHUEGPF) was used as a negative control. Treated cells were harvested 48 hours after transfection for Western blotting and immunofluorescence experiments. shRNA constructs were purchased from Openbiosystems and used to establish stable shRNA expressing HeLa cells following the manufacture's protocol. The following shRNA constructs were used: pGIPZ-WIPI1-2, RHS4430-98853022; pGIPZ-non-targeting control RMS4348.

### Northern blot analysis

Total RNA was extracted from 20 wild type and 20 *fan* mutant embryos at 30 and 50 hpf using standard Trizol protocol for RNA isolation. Northern blot analysis was carried out as described in Freed et al, 2012 [20]. For each sample, 2 µg of RNA was separated by electrophoresis on a 1% agarose/1.25% formaldehyde gel in Tricine/Triethanolamine buffer and transferred to a nylon membrane (Hybond-XL, GE Healthcare). Pre-rRNA species were detected by methylene blue staining and hybridization with a ^32^P-radiolabelled oligonucleotide probe to the 5′ETS: CGAGCAGAGTGGTAGAGGAAGAGAGCTCTTCCTCGCTCA. Quantification of pre-rRNA processing was performed using Image J (National Institutes of Health).

### Apoptosis and cell proliferation analyses

Developmentally staged wild type and *fan* mutant embryos, fixed and processed for cryosectioning, were sectioned at 10 µm. Apoptosis TUNEL assay was performed using the *In situ* cell death detection kit, Fluorescein (Roche Applied Science, Indianapolis, IN, USA). Cell proliferation was assayed with phospho-Histone H3 immunofluorescence analysis, using anti- phospho Histone H3 (Ser10) antibody (Cell Signaling, Danvers, MA) and anti-rabbit goat antibody conjugated with Alexafluor 594 (Life Technologies, Grand Island, NY).

## Supporting Information

Figure S1 **Knockdown efficiency of *wdr43* Morpholino.** (A) Diagram of the *wdr43* reporter construct used to test *wdr43* MO knockdown efficiency. *GFP* (green box) was fused in frame to the 3′ end of a portion of the 5′ end of the *wdr43* cDNA including the 5′ UTR (purple line) and first two exons of *wdr43* gene (purple box). Red line indicates the MO target region. The chimeric gene was driven by the CMV promoter and followed by the 3′ SV40 polyA signal (PA). (B–B″) Fluorescence of shield stage zebrafish embryos injected with the *wdr43*-GFP reporter construct and control MO (CMO) (B, fluorescent microscopy; B′ bright field, B″ merged fluorescent and bright field). (C–C) Lack of fluorescence in shield stage zebrafish embryos injected with *wdr43*-GFP reporter constructs and *wdr43* MO (C, fluorescent microscopy; C′ bright field; C″ merged fluorescent and bright field).
(TIF)

Figure S2 **WISH analyses of *wdr43* MO injected embryos, and p53 MO injected *fan* mutants.** WISH was performed to examine NCC markers *crestin* and *dlx2a* expression in 24 hpf wild type embryos (WT), wild type embryos injected with *wdr43* MO, *fan* mutants, and *fan* mutants injected with −53 MO, as indicated. *wdr43* MO injected embryos exhibited down regulated expression of all NCC markers, similar to that observed in *fan* mutants (arrows). Injection of p53MO into single cell stage *fan* mutants rescued NCC marker gene expression (arrows).
(TIF)

Figure S3 **Subcellular localization of EGFP-tagged wild type or *fan* mutant Wdr43 in zebrafish embryos.** Confocal images taken from 24 hpf old zebrafish embryos injected at single cell stage with EGFP tagged wild type (A1–4, B1–4, C1–4) or *fan* mutant (D1–3, E1–3, F1–3)) *wdr43* mRNA. Co-injection of mCherry tagged zebrafish B23 mRNA was used to label nucleoli (A2, B2, C2, D2, E2, F2). Each row of panels indicates different types of cells imaged in the whole embryo. GFP expression was manually saturated in panels (A4, B4, C4) to reveal the entire nucleus.
(TIF)

Figure S4 **Yeast two hybrid analysis of yeast Wdr43/UTP5 and yeast t-UTP subcomplex proteins.** Primers for full length, and *fan* mutant truncated yeast UTP5 were used to amplify and sublcone these yeast cDNA constructs into pGADT7 vector. The remaining yeast expression constructs were obtained from Dr. S. Baerga. P: permissive medium (-Leu and - Trp). S: selective medium (-Ade, -His, -Leu and -Trp).
(TIF)

Figure S5 **qRT-PCR analysis of *WDR43* mRNA expression in *WDR43* shRNA treated HeLa cells.** The relative expression levels of *WDR43* were normalized to human β-actin gene. Bar graph shows data from three independent experiments.
(TIF)
