# Supplementary figures and images for: Tissue Specific Roles for the Ribosome Biogenesis Factor Wdr43 in Zebrafish Development
Source: PLoS Genet. 2014 Jan 30;10(1):e1004074. doi: 10.1371/journal.pgen.1004074 (PMC3907300; doi:10.1371/journal.pgen.1004074)

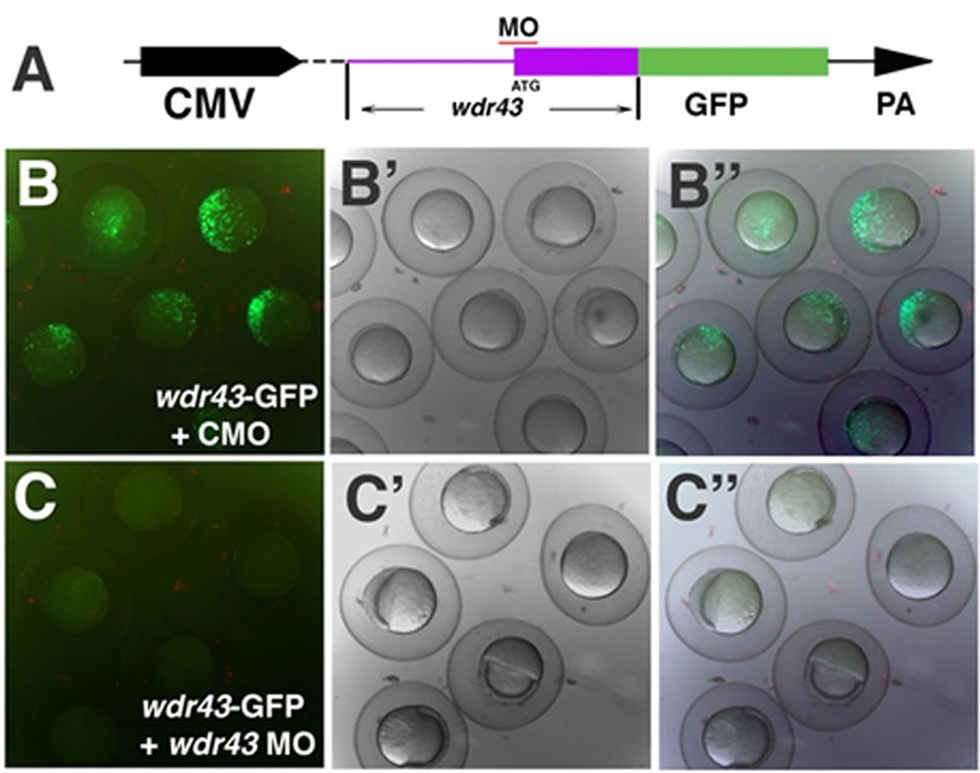

Supplement: Figure S1 — Knockdown efficiency of wdr43 Morpholino. (A) Diagram of the wdr43 reporter construct used to test wdr43 MO knockdown efficiency. GFP (green box) was fused in frame to the 3′ end of a portion of the 5′ end of the wdr43 cDNA including the 5′ UTR (purple line) and first two exons of wdr43 gene (purple box). Red line indicates the MO target region. The chimeric gene was driven by the CMV promoter and followed by the 3′ SV40 polyA signal (PA). (B–B″) Fluorescence of shield stage zebrafish embryos injected with the wdr43-GFP reporter construct and control MO (CMO) (B, fluorescent microscopy; B′ bright field, B″ merged fluorescent and bright field). (C–C) Lack of fluorescence in shield stage zebrafish embryos injected with wdr43-GFP reporter constructs and wdr43 MO (C, fluorescent microscopy; C′ bright field; C″ merged fluorescent and bright field). (TIF) [file pgen.1004074.s001.tif]

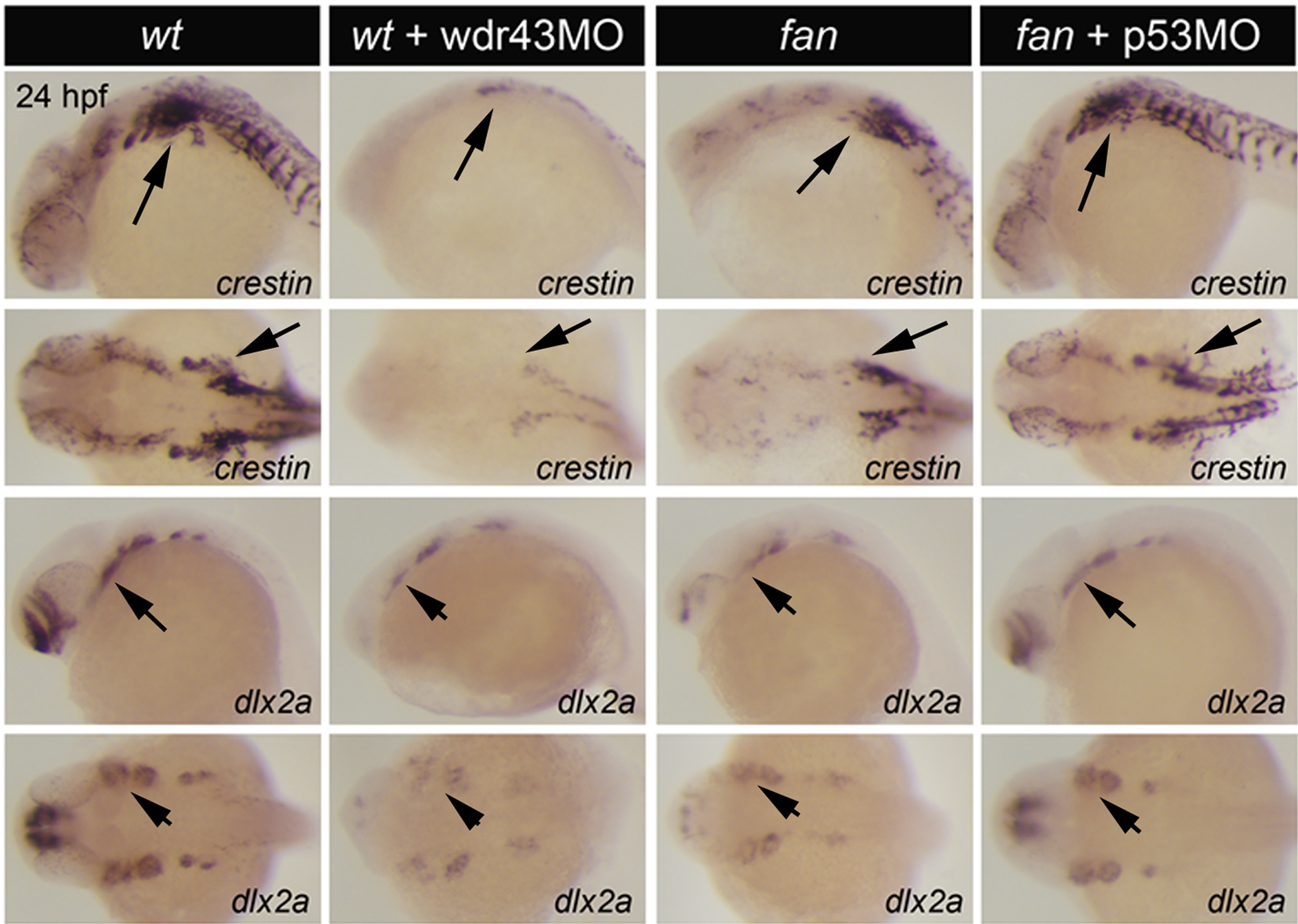

Supplement: Figure S2 — WISH analyses of wdr43 MO injected embryos, and p53 MO injected fan mutants. WISH was performed to examine NCC markers crestin and dlx2a expression in 24 hpf wild type embryos (WT), wild type embryos injected with wdr43 MO, fan mutants, and fan mutants injected with −53 MO, as indicated. wdr43 MO injected embryos exhibited down regulated expression of all NCC markers, similar to that observed in fan mutants (arrows). Injection of p53MO into single cell stage fan mutants rescued NCC marker gene expression (arrows). (TIF) [file pgen.1004074.s002.tif]

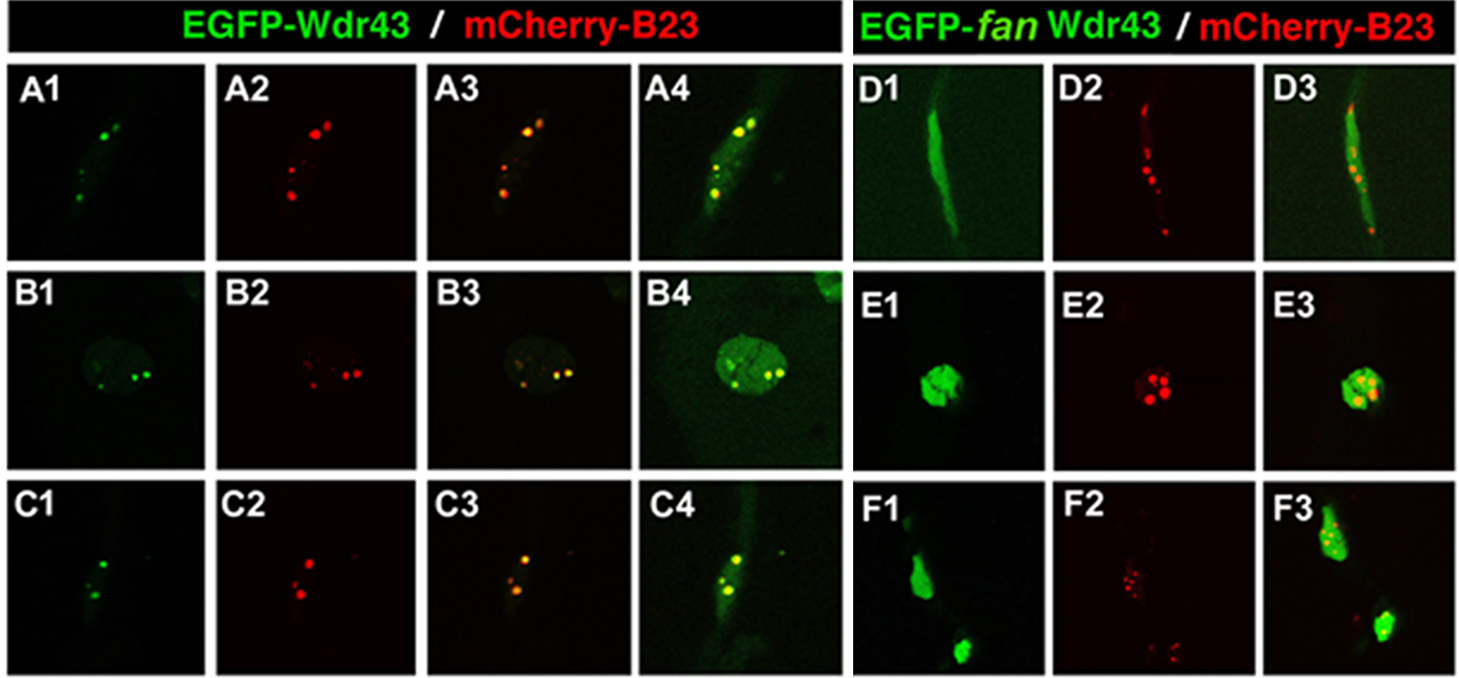

Supplement: Figure S3 — Subcellular localization of EGFP-tagged wild type or fan mutant Wdr43 in zebrafish embryos. Confocal images taken from 24 hpf old zebrafish embryos injected at single cell stage with EGFP tagged wild type (A1–4, B1–4, C1–4) or fan mutant (D1–3, E1–3, F1–3)) wdr43 mRNA. Co-injection of mCherry tagged zebrafish B23 mRNA was used to label nucleoli (A2, B2, C2, D2, E2, F2). Each row of panels indicates different types of cells imaged in the whole embryo. GFP expression was manually saturated in panels (A4, B4, C4) to reveal the entire nucleus. (TIF) [file pgen.1004074.s003.tif]

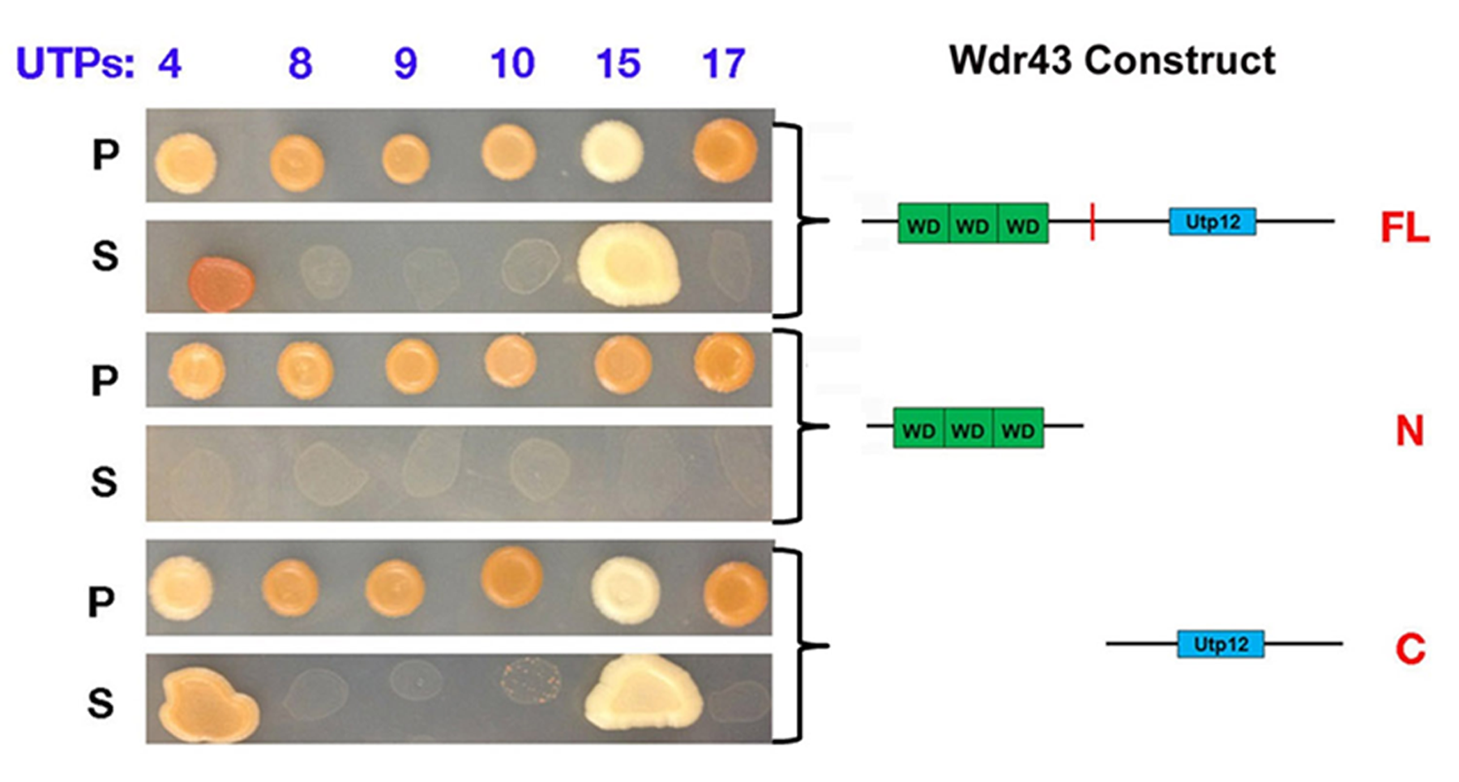

Supplement: Figure S4 — Yeast two hybrid analysis of yeast Wdr43/UTP5 and yeast t-UTP subcomplex proteins. Primers for full length, and fan mutant truncated yeast UTP5 were used to amplify and sublcone these yeast cDNA constructs into pGADT7 vector. The remaining yeast expression constructs were obtained from Dr. S. Baerga. P: permissive medium (-Leu and - Trp). S: selective medium (-Ade, -His, -Leu and -Trp). (TIF) [file pgen.1004074.s004.tif]

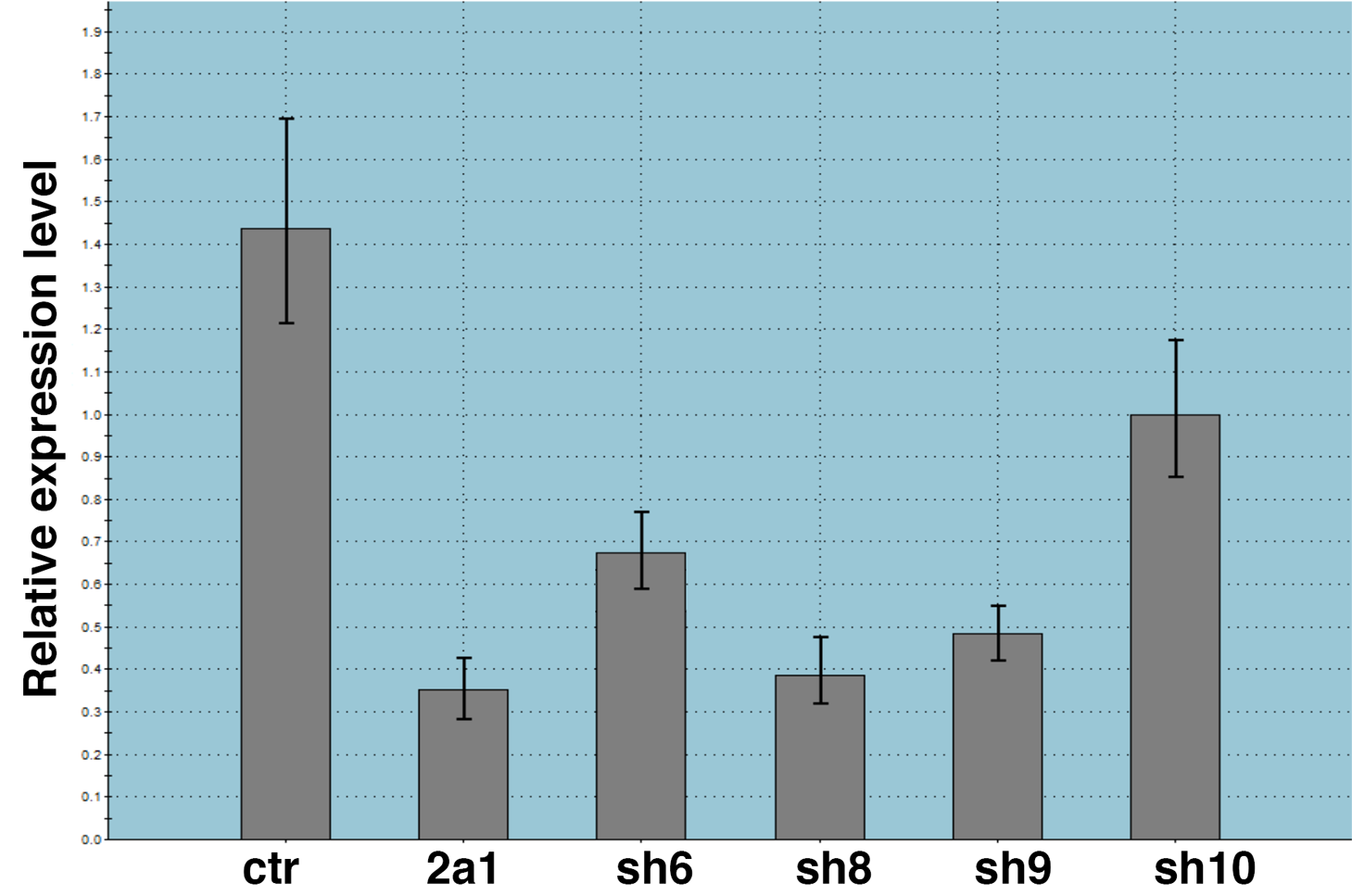

Supplement: Figure S5 — qRT-PCR analysis of WDR43 mRNA expression in WDR43 shRNA treated HeLa cells. The relative expression levels of WDR43 were normalized to human β-actin gene. Bar graph shows data from three independent experiments. (TIF) [file pgen.1004074.s005.tif]
